# The Value of Cystoscopy

**Published:** 1910-02-05

**Authors:** 


### Surgery.

### THE VALUE OF CYSTOSCOPY.

The introduction of the cystoscope and its adoption into general use has produced a change in genito-urinary surgery. To use a simple metaphor, the rule of thumb has been superseded by the rule of science. Previously we had to rely on careful examinations of the constituents of the urine as the principal means of diagnosing which element of the genito-urinary apparatus was primarily at fault. By this means, in conjunction with the clinical examination of the patient, it was usually possible to be moderately certain whether one was dealing with disease of the bladder or the kidneys: but in the latter case it was not always easy to say which kidney was abnormal, since reliance had to be placed on the presence or absence of a renal swelling palpable through the abdominal parietes or of tenderness in one or other subcostal region; and it was a fairly common occurrence to find both these signs absent, and yet on the post-mortem table one or other kidney was discovered to be in an advanced condition of disease. Further, presuming that a diagnosis of disease of one kidney could be made, the methods at our disposal for estimating the adequacy of the other kidney—a point of tremendous importance in the event of nephrectomy being indicated—were both clumsy and inaccurate. In the early days of renal surgery an anterior transperitoneal incision was advocated in order that the opposite kidney might be palpated before any further procedures were undertaken; or as an alternative method it was suggested that the opposite kidney should be explored through a separate incision in the other loin. Both these methods were subject to two obvious fallacies—(1) a kidney which may feel perfectly normal may be performing its functions inadequately, or (2) conversely, a kidney whose contour or size is unusual may be a completely efficient organ: a congenitally lobulated kidney, which is not otherwise abnormal, may be quoted as an instance of the latter. In many cases not even this compromise with the conscience of the operator was undertaken, a diseased kidney being gaily removed on the hypothesis that it was so much diseased as to be entirely useless, and therefore the other one was necessarily adequate for the excretory demands of the individual! A more reasonable suggestion was that a diseased kidney should never be removed primarily, but drained only, so that the urine excreted from it might be discharged from the loin. The urine from the bladder then came from the opposite kidney, whose efficiency could thus be tested by an examination of the amount of urea excreted in the 24 hours. Here again a fallacy existed, because it could never be made certain that all the urine from the drained kidney escaped by the tube and none went down the ureter. The next advance was the invention of Luy's separator. This is an instrument shaped like a large curved bougie, which is introduced into the bladder. In the concavity of the curve there is a diaphragm which can be released by means of a screw until it mechanically divides the bladder into two halves. In the shaft is a two-way channel, so that the urine extruded from each half of the bladder can be examined separately. This is ingenious in theory, but not reliable in practice, since it is practically impossible mechanically to divide the bladder into two compartments so watertight that urine cannot escape from one to the other; and worse than this, it may be positively misleading. For instance, in a case of pyuria, it has been known that the catheter on the diseased side became plugged with pus. The resistance so caused induced all the bladder contents to come from the separator on the side of the sound kidney, after making their way between the diaphragm and the bladder, so that the healthy kidney was suspected of being the diseased one.

The technique of the examination is not difficult. There are now several excellent cystoscopes on the market, and we are not concerned here with their relative merits. The advice we would give is to obtain a cystoscope and become thoroughly acquainted with it. A few practical points may be mentioned. (1) Make sure, by preliminary trial, that the whole apparatus, including the battery, is in working order before the examination is begun. (2) Thoroughly wash out the bladder until the fluid returns quite clear: the slightest turbidity of the bladder contents will impair the view. Leave in a measured quantity of clear fluid: ten ounces of boracic solution will be found sufficient. (3) Have the patient lying on his back with a sandbag under the sacrum. This will bring the bladder forward, and render the examination easier. The lithotomy position produces the reverse result. When all is ready, introduce the cystoscope, as one would a large metal sound, and push it in until the point impinges against the bladder wall. The first points to examine are the ureteric orifices. It will be noticed that on the periphery of the eye-piece there is a small knob. This indicates the position of the curved point of the instrument in which is situated the illumination. After introduction this is uppermost. Rotate it through an angle of 90° to one or other side, and the corresponding ureteric orifice will at once come into view. A normal ureter has a pin-point opening which contracts about once in 20 seconds and discharges a drop of urine. Any abnormality can be observed directly. Thus it may be seen to discharge a white cloud of pus or a dark stream of blood; or it may bulge into the bladder from impaction of a calculus, or be drawn outwards in the shape of a funnel: this is suggestive of a tuberculous kidney. Catheterisation of the ureters is done with a special cystoscope, which carries a long fine catheter. When the ureteric orifice has been located, the point of the catheter is guided by screws which move it in the two planes, and thus it can be introduced along the ureter and the urine from each kidney withdrawn separately and subsequently examined.

